# Global Update on Measles Molecular Epidemiology

**DOI:** 10.3390/vaccines12070810

**Published:** 2024-07-22

**Authors:** Bettina Bankamp, Gimin Kim, Derek Hart, Andrew Beck, Myriam Ben Mamou, Ana Penedos, Yan Zhang, Roger Evans, Paul A. Rota

**Affiliations:** 1 Centers for Disease Control and Prevention, 1600 Clifton Road NE, Atlanta, GA 30329, USA; ofi6@cdc.gov (G.K.); lpu5@cdc.gov (A.B.); 2 ASRT, Inc., Atlanta, GA 30346, USA; ule1@cdc.gov; 3 World Health Organization Regional Office for Europe, 2100 Copenhagen, Denmark; benmamoum@who.int; 4 United Kingdom Health Security Agency, London NW9 5EQ, UK; ana.penedos@ukhsa.gov.uk; 5 WHO Western Pacific Regional Measles/Rubella Reference Laboratory, National Institute for Viral Disease Control and Prevention, Beijing 100013, China; zhangyan9876543@163.com; 6 World Health Organization Western Pacific Regional Office, Manila 1000, Philippines; revans@who.int

**Keywords:** measles, molecular surveillance, elimination, genotyping

## Abstract

Molecular surveillance of circulating measles variants serves as a line of evidence for the absence of endemic circulation and provides a means to track chains of transmission. Molecular surveillance for measles (genotyping) is based on the sequence of 450 nucleotides at the end of the nucleoprotein coding region (N450) of the measles genome. Genotyping was established in 1998 and, with over 50,000 sequence submissions to the Measles Nucleotide Surveillance database, has proven to be an effective resource for countries attempting to trace pathways of transmission. This review summarizes the tools used for the molecular surveillance of measles and describes the challenge posed by the decreased number of circulating measles genotypes. The Global Measles and Rubella Laboratory Network addressed this challenge through the development of new tools such as named strains and distinct sequence identifiers that analyze the diversity within the currently circulating genotypes. The advantages and limitations of these approaches are discussed, together with the need to generate additional sequence data including whole genome sequences to ensure the continued utility of strain surveillance for measles.

## 1. Introduction

**Figure 1:**
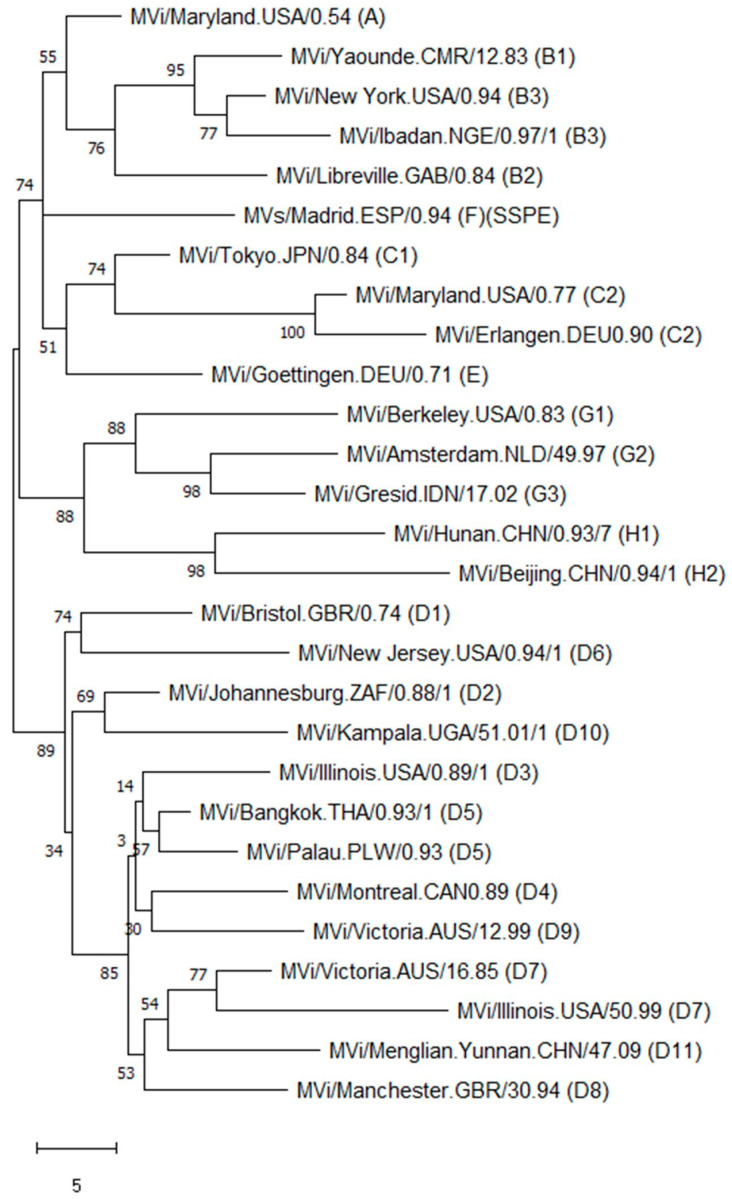
Phylogenetic tree with 28 measles N450 reference sequences. Data from MeaNS [8]. The evolutionary history was inferred using the maximum parsimony method. The percentage of replicate trees in which the associated taxa clustered together in the bootstrap test (500 replicates) are shown next to the branches. The tree is drawn to scale, with branch lengths calculated using the average pathway method and are in the units of the number of changes over the whole sequence. Scale bar indicates number of nucleotides.

Member states in all six regions of the World Health Organization (WHO) have declared measles elimination goals [1]. Molecular surveillance of circulating measles variants serves as a major line of evidence for the absence of endemic circulation and provides support for the classification of cases as imported, import-related or endemic [2,3]. Based on the sequences of their hemagglutinin and nucleocapsid (N) gene-coding sequences, measles viruses have been divided into 24 genotypes [4,5,6,7]. Specimens from new cases are assigned to one of these genotypes by sequencing the 450 nucleotides at the end of the nucleoprotein gene coding region (N450) and comparing these to a set of established reference sequences (Figure 1).

Standard molecular surveillance for measles based on N450 sequencing was established in 1998 [4]; these sequences are submitted to the Measles Nucleotide Surveillance (MeaNS) database which holds >59,000 sequences [8]. Every N450 sequence receives a name (“WHO name”) which includes information about the place and time of the case [4]. For example, MVs/Georgia.USA/10.24 would be the sequence name for a case in the state of Georgia, USA in epidemiological week 10 of 2024. The WHO names link the sequence information to epidemiological data. The Global Measles and Rubella Laboratory Network (GMRLN) in the WHO has standardized naming conventions in a series of publications [4,5,7,9,10]. Countries with endemic measles are encouraged to provide sequences from large outbreaks to generate baseline data that can be used for comparison once these countries get closer to elimination status. Countries in or near elimination status should obtain genotype information from 80% of all chains of transmission, including one case chains [2].

Through the International Reagent Resource (IRR, [11]) the GMRLN offers kits for Sanger sequencing of N450 and protocols are available from the website of the WHO measles and rubella laboratory manual [12]. Unlike other pathogens, which use sequencing to monitor the development of vaccine or drug escape mutants or to identify candidate strains for updated vaccines [13,14,15,16,17,18,19], the major purpose of sequencing measles specimens is to monitor viral transmission and progress toward elimination. Since even a few nucleotide changes may affect the interpretation of sequencing data, the GMRLN has a strong emphasis on sequence quality. Efforts to improve and monitor the quality of molecular testing in laboratories of the GMRLN are described elsewhere [20]. This review describes the reduced diversity of circulating genotypes and the approaches developed by the GMRLN to replace the comparison between genotypes with the interpretation of sequence differences within genotypes. Limitations to the use of the small standard genotyping window, options for extended sequencing and obstacles to the widespread introduction of extended sequencing are discussed.

## 2. Reduced Diversity of Circulating Genotypes

**Figure 2:**
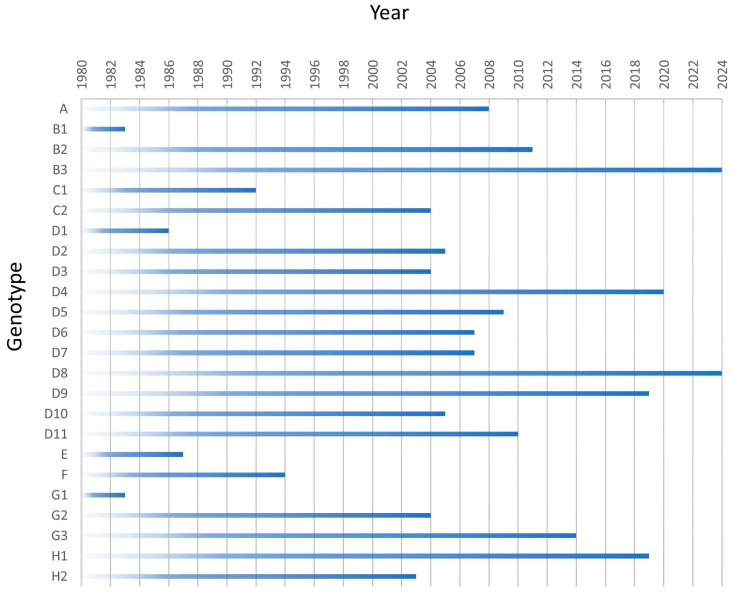
Year of last detection of measles genotypes. The last year of documented circulation reported to MeaNS [8] for all 24 genotypes is shown. Viruses with a date of 2024 are currently circulating. All other viruses have had transmission interrupted in the year depicted. Note that the chart does not specify the first year of detection of any genotype.

**Figure 3:**
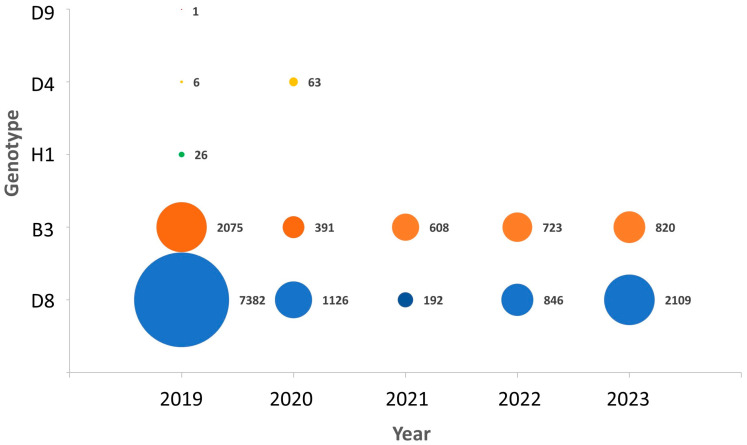
Number of submissions per genotype reported to MeaNS [8] 2019–2023.

Eighteen co-circulating genotypes were detected in 2003 (Figure 2). The number of genotypes has decreased steadily since then and only two genotypes, B3 and D8, have been detected since 2021 (Figure 2 and Figure 3).

A caveat to these observations is that the quality of molecular surveillance varies between countries and regions; major surveillance gaps exist in the African, Southeast Asian and Eastern Mediterranean regions. Table 1 shows submissions to MeaNS per WHO region; comparing these to the numbers of estimated cases [21,22,23,24] demonstrates the discrepancy between case numbers and genotyping information. Estimated case numbers were not yet available for 2023. The table covers the years of the pandemic and shows that all WHO regions faced difficulties in maintaining molecular surveillance for measles in 2020 and 2021. However, in 2022, the ratio between estimated cases and MeaNS submissions had largely recovered to pre-pandemic levels. 

While the GMRLN is cautious about declaring genotypes as inactive, it is clear that the number of circulating genotypes has been reduced, suggesting that vaccination efforts have interrupted the transmission of several genotypes. A recent example is the disappearance of genotype H1, which has been endemic in the People’s Republic of China (PRC) since the late 1990s [25]. While genotype H1 was frequently exported from the PRC, it never established sustained continued transmission anywhere else. Highly successful vaccination efforts in the PRC appear to have ended circulation of genotype H1 in the country. In 2018, 275 of 316 submissions to MeaNS were genotype H1; in 2019, there were only 21 genotype H1 reports in 427 submissions and the last case with an H1 genotype was reported to MeaNS in September 2019 [8,26]. The last detection of genotype H1 in the PRC corresponds with the failure to detect genotype H1 associated with imported cases globally.

## 3. Utilizing the Diversity within Genotypes

**Figure 4:**
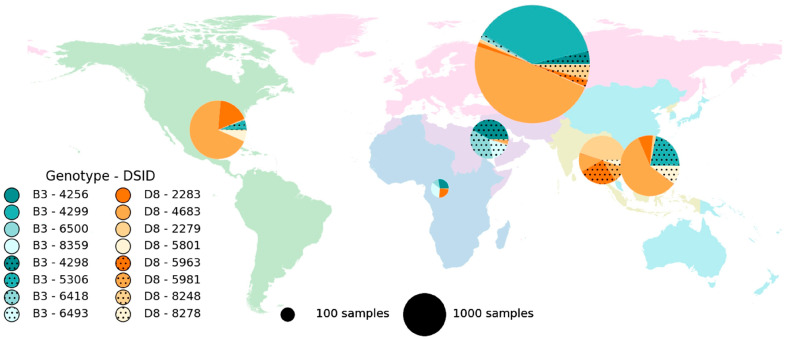
Global distribution of major DSIds according to the WHO region, 2019–2023. The genotype is indicated by the color and pattern in each pie chart. The size of the pie chart indicates the number of submissions to MeaNS [8]. Only the eight most frequently detected DSIds are listed.

The decrease in the number of circulating genotypes reduced the utility of N450-based genotyping for tracing the pathways of transmission since all sequences are now classified as genotype B3 or D8. However, there is sequence diversity within genotypes and the GMRLN has formalized approaches to characterize this diversity. One tool is the use of a distinct sequence identifier (DSId) [10]. Every novel sequence submitted to MeaNS receives a unique DSId. All submissions of identical sequences receive the same DSId. A MeaNS user with a new sequence can search MeaNS to find identical sequences, i.e., sequences that have the same DSId. The detection of identical sequences in other countries can then be used to support epidemiological data on the import status of a measles case. Different DSIds are prevalent in different parts of the world (Figure 4), indicating the utility of DSIds compared to using genotypes alone.

Information about the detection of DSIds in other countries as well as the global distribution of the DSId should be added to reports to national or regional verification commissions (NVC, RVC) [10], which annually examine the elimination status of countries.

**Figure 5:**
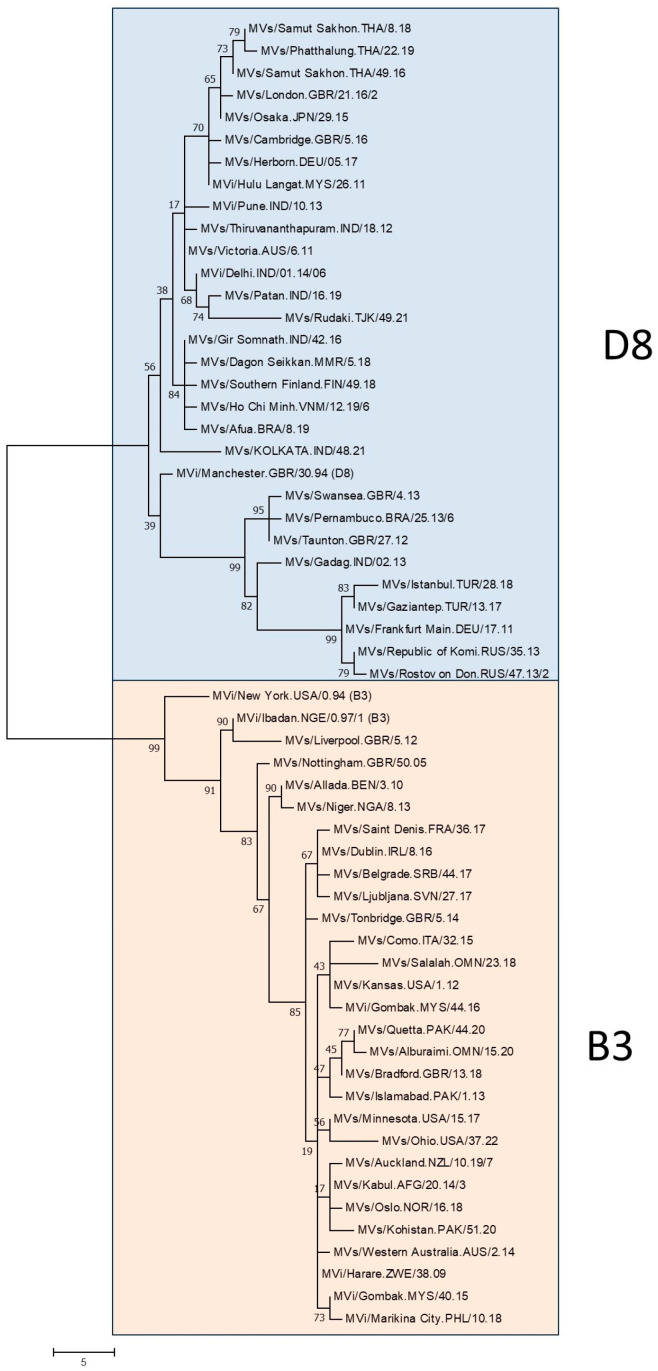
Phylogenetic tree of named strains of genotypes B3 and D8. Data from MeaNS [8], accessed 9 April 2024. The percentage of replicate trees in which the associated taxa clustered together in the bootstrap test (500 replicates) are shown next to the branches. The tree is drawn to scale, with branch lengths calculated using the average pathway method and are in the units of the number of changes over the whole sequence. Scale bar indicates number of nucleotides.

**Figure 6:**
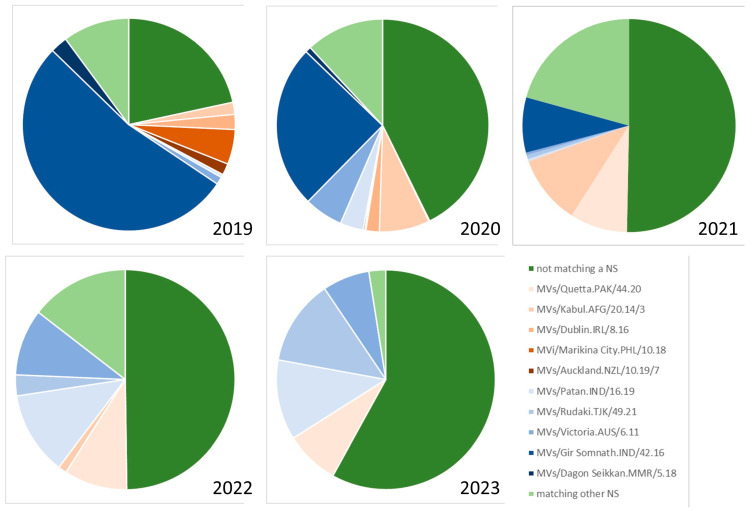
Proportion of MeaNS submissions corresponding to the five most frequently reported named strains of genotypes B3 (shades of orange) and D8 (shades of blue), 2019–2023. Data from MeaNS [8], accessed 9 April 2024. NS = named strain.

A second tool is the use of named strains (Figure 5). Named strains are identical sequences (i.e., submissions with the same DSId) that have been submitted to MeaNS at least 50 times from multiple countries over the course of the previous two years [9]. Named strains are variants with epidemiological importance as they are widely distributed. The WHO name of the first submission to MeaNS with the relevant N450 sequence is used to identify the named strain. For example, a sequence variant that was found globally in 2018/2019 was MVs/Gir Somnath.IND/42.16, with >6500 submissions to MeaNS. The name of the strain cannot be used as an indication of the source country of an importation, since the very existence of the named strain indicates that this variant is present in multiple countries. Linking a sequence to a named strain in MeaNS implies that the new sequence is part of a lineage with global distribution, which is easier than listing a large number of possible import sources. Figure 6 shows the proportion of the most frequently identified named strains for B3 and D8 from 2019 to 2023, demonstrating the changes in the most commonly found variants over time. Interestingly, the proportion of submitted sequences that are not identical to a named strain (dark green in Figure 6) has continually increased since 2019 and now makes up more than half of all submissions. This may indicate continuing diversification withing the two genotypes or it may be an artifact of surveillance. For example, if a country with highly active surveillance sequences many specimens from a large outbreak of a named strain, those sequences will make up a relatively large proportion of all submitted sequences.

**Figure 7:**
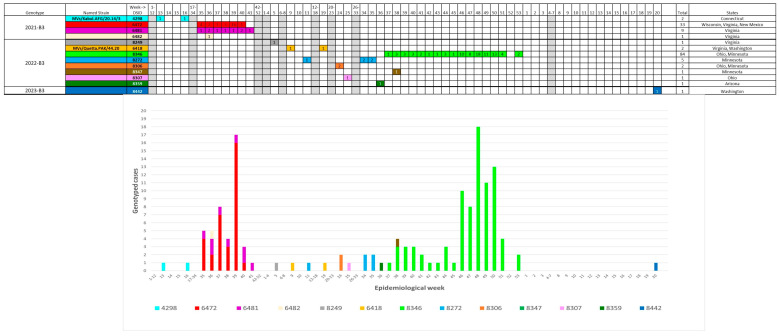
Example for reporting to NVC [27]: Genotype B3 cases in the US 2021–2023. (**Top**) Chart format. Columns represent epidemiological weeks. Columns in grey are combined weeks without sequenced cases. The number in each cell indicates the number of identical sequences from cases with onset in that week. (**Bottom**) Curve format. The data and colors are the same as on top. Colors correspond to different DSIds.

Visualization tools for reports to the NVC or RVC include charts to display DSIds detected by epidemiologic week (Figure 7). The WHO Regional Office for Europe developed an approach that brings together on a single visual all the information about the chains of transmission and sporadic cases in a given country over time with their respective DSIds/named strains, in combination with the epidemiological curve and the information about importations, using data from the NVC’s annual report (Annual Status Update) and MeaNS. This approach proved to be a very useful tool to support the decision-making process of the European RVC (Supplementary Figure S1).

Since the initiation of standard protocols for genotyping, adding the genotype information to the WHO name served to describe genetic relatedness between sequences, but the reduction in diversity of circulating viruses now requires a different approach. Efforts are under way to define epidemiologically relevant lineages within genotypes B3 and D8 which will serve as the basis for an updated nomenclature. This nomenclature is expected to maintain the WHO name but add concise information about lineage assignment.

## 4. Options for Extended Sequencing

Within a genotype, many sequences are identical to named strains, which are of limited utility for molecular surveillance because of their wide circulation. While N450 sequencing remains the standard in the GMRLN, the resolution offered by a 450-nucleotide window is limited and extended sequencing approaches are required. To provide equitable options to countries needing molecular data for elimination reports, extended sequencing efforts in the GMLRN must consider the varying levels of technical capacity in national laboratories and the funding options for lower-income countries.

**Figure 8:**
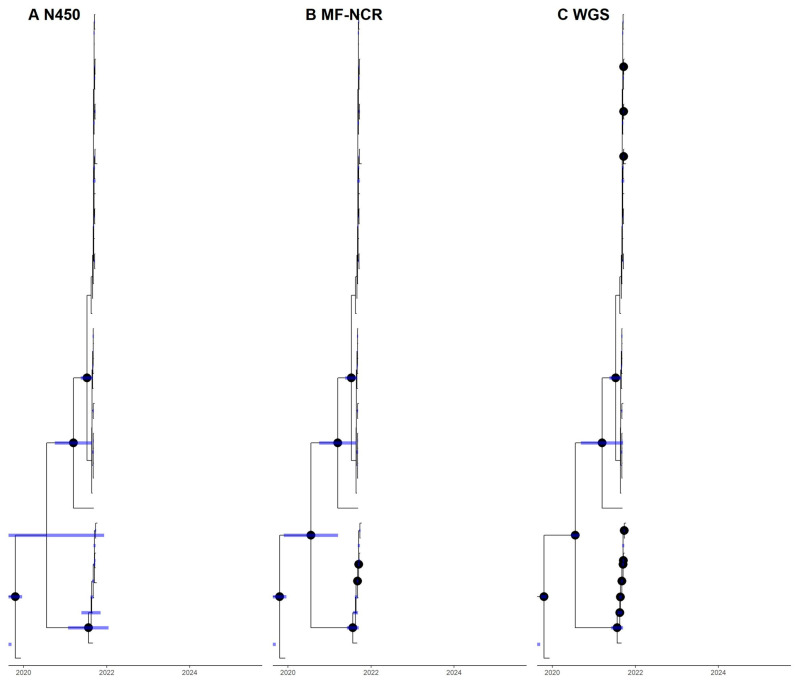
Comparison of N450, MF-NCR and WGS. BEAST analysis of (**A**) N450 sequences, (**B**) MF-NCR sequences and (**C**) WGS of the same samples. *X*-axis indicates time scale. Purple bars indicate 95% confidence interval for time points of branching patterns. Black dots indicate high statistical support for branch points. Analysis of longer sequences improves statistical support for branching patterns and narrows confidence intervals. Data are based on published sequences [28], but annotations are simulated to show how the certainty of the tree shape and measurements are likely to change.

Consequently, laboratories have two options for obtaining additional sequence information from measles cases: sequencing the non-coding region between the matrix and fusion protein coding regions (MF-NCR) or whole genome sequencing (WGS). At 1012 nucleotides in length, the MF-NCR is the only long non-coding part and one of the most variable regions of the measles genome. Generally, the acquisition of longer sequence regions or regions of high intratypic diversity provides a better estimate of the substitution patterns along the measles virus (MeV) genome; comparison studies demonstrate that sequencing the MF-NCR offers improved phylogenetic model resolution over N450, especially regarding the statistical support of branching patterns. When molecular clock models are used, the MF-NCR in many cases produces greater certainty (narrower confidence intervals) for posterior estimates of internal node dating (Figure 8). Through the IRR, the GMRLN distributes a kit for Sanger sequencing the MF-NCR to support sequencing in low-income countries and a protocol is available from the WHO Lab Manual website [12].

**Figure 9:**
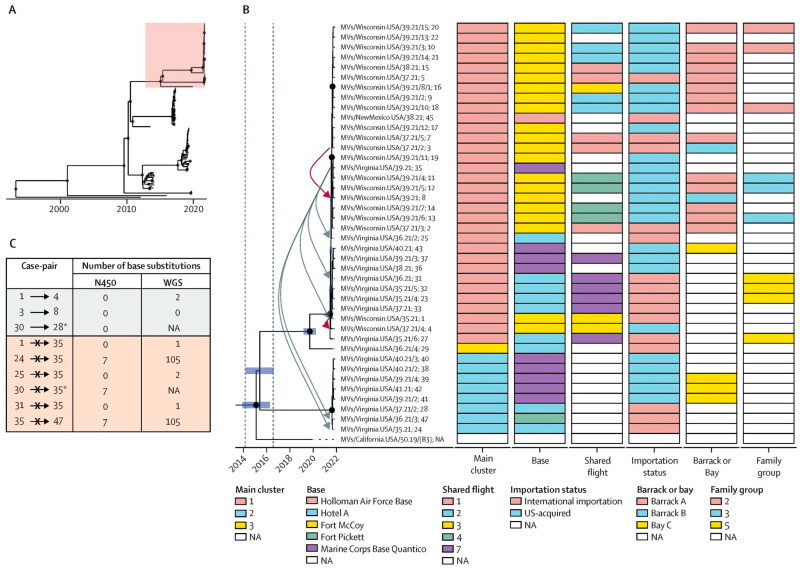
From [28], with permission of the authors. Use of WGS in combination with epidemiological data. Measles outbreak among Afghan refugees in the USA in 2021. (**A**) Red square indicates part of tree magnified in B. (**B**) *X*-axis indicates time. Blue bars denote 95% confidence intervals. Red arrows highlight epidemiologically linked cases. Grey arrows highlight cases without epidemiological links. (**C**) High number of nucleotide differences can rule out epidemiological linkage but low numbers do not always serve as proof of linkage. * WGS not available.

WGS provides the most complete reconstructions of MeV evolution; however, it is currently only conducted in a small number of laboratories in the GMRLN. The utility of analysis of WGS has been demonstrated for the analysis of a number of outbreaks. A recent example was the analysis of measles cases observed in Afghan evacuees resettled in the USA following the fall of Kabul, in 2021 (Figure 9 [28]). Methods utilizing well-accepted next-generation sequencing (NGS) platforms (Illumina, Oxford Nanopore Technologies (ONT)) have been developed by several laboratories in the GMRLN. At present, the GMRLN has limited resources to support the widespread implementation of NGS and does not offer protocols or reagents. NGS may be initiated with a regional approach with regional reference laboratories supporting national laboratories.

Ensuring high-quality sequencing results is more difficult when using NGS methods, due to the complex, multistep nature of the methods and the greater variability in technical approaches. The GMRLN is finalizing a guidance document for quality control of NGS, for both the laboratory and the bioinformatics methods. There are considerable obstacles to the widespread adoption of WGS bioinformatics methods within the GMRLN laboratories. Chiefly, the computational infrastructure required for institutional-grade analyses of NGS data exceeds the capacity of many national laboratories, with respect to both equipment and available bioinformatics expertise. Several laboratories in the GMRLN are in the process of developing sequence analysis pipelines to automate NGS analyses according to the requirements of MeV surveillance. Ideally, these pipelines would be shared or made accessible to contributors from outside the institutions where the pipelines are developed.

The GMRLN has developed guidelines to support decision-making by national laboratories and regional coordinators concerning the need for extended sequencing, whether it is MF-NCR sequencing or WGS [12]. The objective is to recommend extended sequencing to countries in or near elimination where it is expected to bring an added value compared to N450 sequencing. This approach will make a difference for the verification of elimination and provide countries without the capacity for extended sequening access a regional sequencing center.

## 5. Options for Interpreting Sequencing Data

For many years, the standard approach to interpret N450 sequences has been to generate phylogenetic trees that infer relative genetic distances (Figure 1). Capacity building within the GMRLN has led to an increased understanding and use of bootstrap values to evaluate the statistical significance of the results of phylogenetic analysis. However, since the goal of sequencing measles specimens is to determine whether cases are part of the same transmission chain or the result of separate importations, genetic distance alone is ultimately not sufficient to interpret the relevance of a small number of nucleotide changes. One well-accepted software platform for time-based phylogenetic analyses is Bayesian evolutionary analysis by sampling trees (BEAST) [29]. Bayesian tree inference is increasingly popular, accommodating inference of divergence time alongside that of nucleotide substitution patterns. BEAST uses time as the *X*-axis and inferences can be based on N450 sequences, MF-NCR sequences and WGS (Figure 8 and Figure 9). Bayesian analyses require a large, curated dataset, considerable computational resources, and bioinformatics expertise. An alternative probabilistic approach simplifies the comparison of two sequences [30]. However, it requires reasonable assumptions about the most recent common ancestor of the sequences under comparison and its implementation would require standard nucleotide substitution rates (molecular clocks).

## 6. Molecular Surveillance Supports Development of Diagnostic Assays and Vaccines

While outside the scope of this review, it is worth mentioning that the molecular surveillance of measles viruses generates data for other activities that are essential for measles elimination. First, it provides supporting data to ensure continued functionality of molecular diagnostic assays. Through the IRR, the GMRLN distributes a kit for real-time RT-PCR (rRT-PCR) that is used in many countries for the detection of measles RNA in clinical specimens. The primers and probe for this rRT-PCR assay bind within the standard genotyping window (N450); hence, all genotyping data also monitor the genetic stability of the rRT-PCR primer and probe binding sites. A recent publication [31] identified a lineage of measles virus with nucleotide substitutions in the binding site for the reverse primer of the rRT-PCR assay which led to reduced sensitivity of the diagnostic assay. Consequently, the rRT-PCR reverse primer was modified to restore sensitivity for this measles lineage [32]. While the primers and probe used in the IRR kit are widely used in the GMRLN, many laboratories use other molecular diagnostic assays whose primers and probe bind outside of N450. The increased use of WGS will provide data to monitor the genetic stability of those diagnostic assays.

Secondly, molecular surveillance provides supporting data to monitor the effect of viral evolution on vaccine efficacy. Measles has only one serotype and the vaccine strains developed decades ago still protect against the measles lineages circulating today. However, as an RNA virus, measles is subject to more rapid molecular evolution than DNA viruses [33]. This genetic drift may change epitopes, the protein sequences that are recognized by the immune system, on measles proteins, which could lead to reduced efficacy of the vaccines [34]. WGS provides data for ongoing monitoring of the evolution of immunologically relevant epitopes, and, if necessary, will provide nucleotide sequence data for the development of updated measles vaccines.

## 7. Limitations and Outlook

Any analysis of measles variants is affected by the sequence surveillance gaps present in multiple countries and regions. These gaps widened during the COVID-19 pandemic and surveillance has not yet recovered everywhere. It is expected that this situation will gradually improve as more countries approach elimination; however, surveillance also needs to improve in countries with endemic measles as these are the most frequent sources of imported virus. One reason for the surveillance gaps is a lack of specimens for viral detection and genotyping in many countries. Many national laboratories are proficient in molecular methods, but either do not receive specimens or do not receive the appropriate specimen type. Serology remains the most commonly used method for case confirmation and the collection of throat swabs, nasopharyngeal swabs or urine samples for molecular assays is still not routine in many countries. For example, in the first half of 2023, 33 countries in the European region reported measles cases, but only 18 countries submitted sequences to MeaNS [35]. This is largely due to lack of specimens, as all countries in the European region have access to genotyping, either through their national laboratory or a regional reference laboratory. Improving specimen collection requires training for surveillance staff and program managers as well as the availability of specimen collection kits and adequate specimen shipping and storage.

Adjustments to address the challenge posed by the reduced diversity of circulating viruses have only just begun and will require additional resources, not only for equipment, training, and reagents for extended sequencing but also for staffing to manage the workload. Of course, since extended sequencing has begun only recently, surveillance gaps for MF-NCR sequences and WGS are even larger than the gaps for N450 sequences. Using extended sequencing to trace pathways of transmission is only useful if representative sequences from all countries are available in the database. The COVID-19 pandemic has led to a large increase in NGS sequencing capacity in many laboratories. The challenge for the GMRLN is to leverage this capacity to generate high-quality measles sequences. Establishing regional sequencing centers and developing shared sequencing pipelines will be required to meet the challenge.

Sequencing data cannot substitute for missing epidemiological information. Even WGS cannot distinguish between repeated importations into country A from the same outbreak in country B versus continuous circulation in country A. Epidemiological surveillance and collaboration between laboratory scientists and epidemiologists need to be improved to generate high-quality data for national programs.

## 8. Conclusions

Over more than two decades, the GMRLN has built an effective strain surveillance system that has provided sequence data to monitor pathways of transmission and support for verification of measles elimination. The challenge posed by the decreased number of circulating measles genotypes was addressed by tools that analyze the diversity within the currently circulating genotypes. However, the continued utility of strain surveillance will require the introduction of extended sequencing approaches. While acknowledging the constraints on capacity and funding in national laboratories, the judicious use of existing referral systems and the appropriate introduction of technology to countries, where required, will maintain and develop the strain surveillance system already being provided by the global laboratory network to address the future needs of measles and rubella elimination.

## Figures and Tables

**Table 1:** Estimated cases compared to number of MeaNS submissions, 2019–2023.

	African Region			American Region			Eastern Mediterranean Region		
Year	Cases *	MeaNS ^#^	Ratio ^	Cases	MeaNS	Ratio	Cases	MeaNS	Ratio
2019	4,548,000	55	82,691	102,700	1397	74	1,384,500	216	6410
2020	1,944,700	17	114,394	43,700	315	139	2,043,600	121	16,889
2021	4,370,190	12	364,183	3410	205	17	2,303,170	538	4281
2022	5,138,698	55	93,431	825	108	8	1,193,257	474	2517
	**European Region**			**South-East Asian Region**			**Western Pacific Region**		
**Year**	**Cases**	**MeaNS**	**Ratio**	**Cases**	**MeaNS**	**Ratio**	**Cases**	**MeaNS**	**Ratio**
2019	494,600	4356	114	2,655,000	942	2818	643,700	2530	254
2020	179,600	473	380	2,552,600	556	4591	784,900	128	6132
2021	86,190	29	2972	1,702,699	13	130,977	958,395	3	319,465
2022	63,707	133	479	1,896,917	780	2432	938,883	19	49,415

* Estimated cases [21,22,23,24]; ^#^ MeaNS submissions [8]; ^ number of estimated cases per MeaNS submission.

## Supplementary Materials

The following supporting information can be downloaded at: https://www.mdpi.com/article/10.3390/vaccines12070810/s1, Figure S1: Example for reporting to NVC, Spain 2018–2019. Top: Epidemiological curve. Bars indicate the number of cases per month. Bottom: lineages including non-genotyped sporadic cases. Columns represent weeks from week 1, 2018 to week 52, 2019. Rows are different DSIds or named strains. The genotype is indicated with each DSID. Dashed cells indicate imported case, solid cells represent chains of transmission. The pattern for D8-Gir Somnath sporadic cases indicates a range of time during which several cases not linked to known chains of transmission occurred.

## References

[1] Measles Overview. https://www.who.int/health-topics/measles.

[2] World Health Organization (2010). Monitoring progress towards measles elimination. Wkly. Epidemiol. Rec..

[3] World Health Organization (2018). Guidance for evaluating progress towards elimination of measles and rubella. Wkly. Epidemiol. Rec..

[4] World Health Organization (1998). Standardization of the nomenclature for describing the genetic characteristics of wild-type measles viruses. Wkly. Epidemiol. Rec..

[5] World Health Organization (2003). Update of the nomenclature for describing the genetic characteristics of wild-type measles viruses: New genotypes and reference strains. Wkly. Epidemiol. Rec..

[6] World Health Organization (2006). Global distribution of measles and rubella genotypes-update. Wkly. Epidemiol. Rec..

[7] World Health Organization (2012). Measles virus nomenclature update: 2012. Wkly. Epidemiol. Rec..

[8] Measles Nucleotide Surveillance (MeaNS). https://who-gmrln.org/means2.

[9] World Health Organization (2015). Genetic diversity of wild-type measles viruses and the global measles nucleotide surveillance database (MeaNS). Wkly. Epidemiol. Rec..

[10] Williams D., Penedos A., Bankamp B., Anderson R., Hübschen J., Mamou M.B., Beck A., Brown D., Rey-Benito G., Evans R. (2022). Update: Circulation of active genotypes of measles virus and recommendations for use of sequence analysis to monitor viral transmission. Wkly. Epidemiol. Rec..

[11] IRR International Reagent Resource. https://www.internationalreagentresource.org/.

[12] (2018). Manual for the Laboratory-Based Surveillance of Measles, Rubella, and Congenital Rubella Syndrome, 3rd ed. https://www.technet-21.org/en/topics/programme-management/manual-for-the-laboratory-based-surveillance-of-measles-rubella-and-congenital-rubella-syndrome/manual-for-the-laboratory-based-surveillance-of-measles-rubella-and-congenital-rubella-syndrome.

[13] Ávila-Ríos S., Parkin N., Swanstrom R., Paredes R., Shafer R., Ji H., Kantor R. (2020). Next-Generation Sequencing for HIV Drug Resistance Testing: Laboratory, Clinical, and Implementation Considerations. Viruses.

[14] Bandera A., Gori A., Clerici M., Sironi M. (2019). Phylogenies in ART: HIV reservoirs, HIV latency and drug resistance. Curr. Opin. Pharmacol..

[15] Deyde V.M., Gubareva L.V. (2009). Influenza genome analysis using pyrosequencing method: Current applications for a moving target. Expert Rev. Mol. Diagn..

[16] McGinnis J., Laplante J., Shudt M., George K.S. (2016). Next generation sequencing for whole genome analysis and surveillance of influenza A viruses. J. Clin. Virol..

[17] Munyuza C., Ji H., Lee E.R. (2022). Probe Capture Enrichment Methods for HIV and HCV Genome Sequencing and Drug Resistance Genotyping. Pathogens.

[18] Van Poelvoorde L., Vanneste K., De Keersmaecker S.C., Thomas I., Van Goethem N., Van Gucht S., Saelens X., Roosens N.H. (2022). Whole-Genome Sequence Approach and Phylogenomic Stratification Improve the Association Analysis of Mutations with Patient Data in Influenza Surveillance. Front. Microbiol..

[19] Zhao X.N., Zhang H.-J., Li D., Zhou J.-N., Chen Y.-Y., Sun Y.-H., Adeola A.C., Fu X.-Q., Shao Y., Zhang M.-L. (2020). Whole-genome sequencing reveals origin and evolution of influenza A(H1N1)pdm09 viruses in Lincang, China, from 2014 to 2018. PLoS ONE.

[20] Bankamp B., Anderson R., Hao L., Chen M., Kim G., Mori Y., Rota P.A. (2024). Building quality control for molecular assays in the global measles and rubella laboratory network. Vaccines.

[21] Patel M.K. (2020). Progress toward Regional Measles Elimination—Worldwide, 2000–2019. MMWR Morb. Mortal. Wkly. Rep..

[22] Dixon M.G. (2021). Progress toward Regional Measles Elimination—Worldwide, 2000–2020. MMWR Morb. Mortal. Wkly. Rep..

[23] Minta A.A. (2022). Progress toward Regional Measles Elimination—Worldwide, 2000–2021. MMWR Morb. Mortal. Wkly. Rep..

[24] Minta A.A. (2023). Progress toward Measles Elimination—Worldwide, 2000–2022. MMWR Morb. Mortal. Wkly. Rep..

[25] Xu W., Tamin A., Rota J.S., Zhang L., Bellini W.J., Rota P.A. (1998). New genetic group of measles virus isolated in the People’s Republic of China. Virus Res..

[26] Wang H., Zhu Z., Duan X., Song J., Mao N., Cui A., Wang C., Du H., Wang Y., Li F. (2023). Transmission Patterns of Measles Virus Circulating in China During 1993-2021: Genotyping Evidence Supports that China is Approaching Measles Elimination. Clin. Infect. Dis..

[27] Mathis D.A. (2024). Measles—United States, 1 January 2020–March 28 2024. MMWR Morb. Mortal. Wkly. Rep..

[28] Masters N.B., Beck A.S., Mathis A.D., Leung J., Raines K., Paul P., E Stanley S., Weg A.L., Pieracci E.G., Gearhart S. (2023). Measles virus transmission patterns and public health responses during Operation Allies Welcome: A descriptive epidemiological study. Lancet Public Health.

[29] Bouckaert R., Vaughan T.G., Barido-Sottani J., Duchêne S., Fourment M., Gavryushkina A., Heled J., Jones G., Kühnert D., De Maio N. (2019). BEAST 2.5: An advanced software platform for Bayesian evolutionary analysis. PLoS Comput. Biol..

[30] Penedos A.R., Fernández-García A., Lazar M., Ralh K., Williams D., Brown K.E. (2022). Mind your Ps: A probabilistic model to aid the interpretation of molecular epidemiology data. EBioMedicine.

[31] Pérez-Rodríguez F.J., Cherpillod P., Thomasson V., Vetter P., Schibler M. (2024). Identification of a measles variant displaying mutations impacting molecular diagnostics, Geneva, Switzerland, 2023. Eurosurveillance.

[32] Beck A.S., Lopareva E.N., Hwang H., Hart D., de Almeida M., Anderson R., Rota P.A., Bankamp B. (2024). Evaluation of the Sensitivity of a Measles Diagnostic RT-qPCR Assay Incorporating Recently Observed Priming Mismatch Variants. Euro Surveill..

[33] Peck K.M., Lauring A.S. (2018). Complexities of Viral Mutation Rates. J. Virol..

[34] Tahara M., Ito Y., Brindley M.A., Ma X., He J., Xu S., Fukuhara H., Sakai K., Komase K., Rota P.A. (2013). Functional and structural characterization of neutralizing epitopes of measles virus hemagglutinin protein. J. Virol..

[35] WHO European Region (2023). WHO EpiBrief: A Report on the Epidemiology of Selected Vaccine-Preventable Diseases in the European Region: No. 2/2023.

