# Supplementary figures and images for: Global Update on Measles Molecular Epidemiology
Source: Vaccines (Basel). 2024 Jul 22;12(7):810. doi: 10.3390/vaccines12070810 (PMC11281501; doi:10.3390/vaccines12070810)

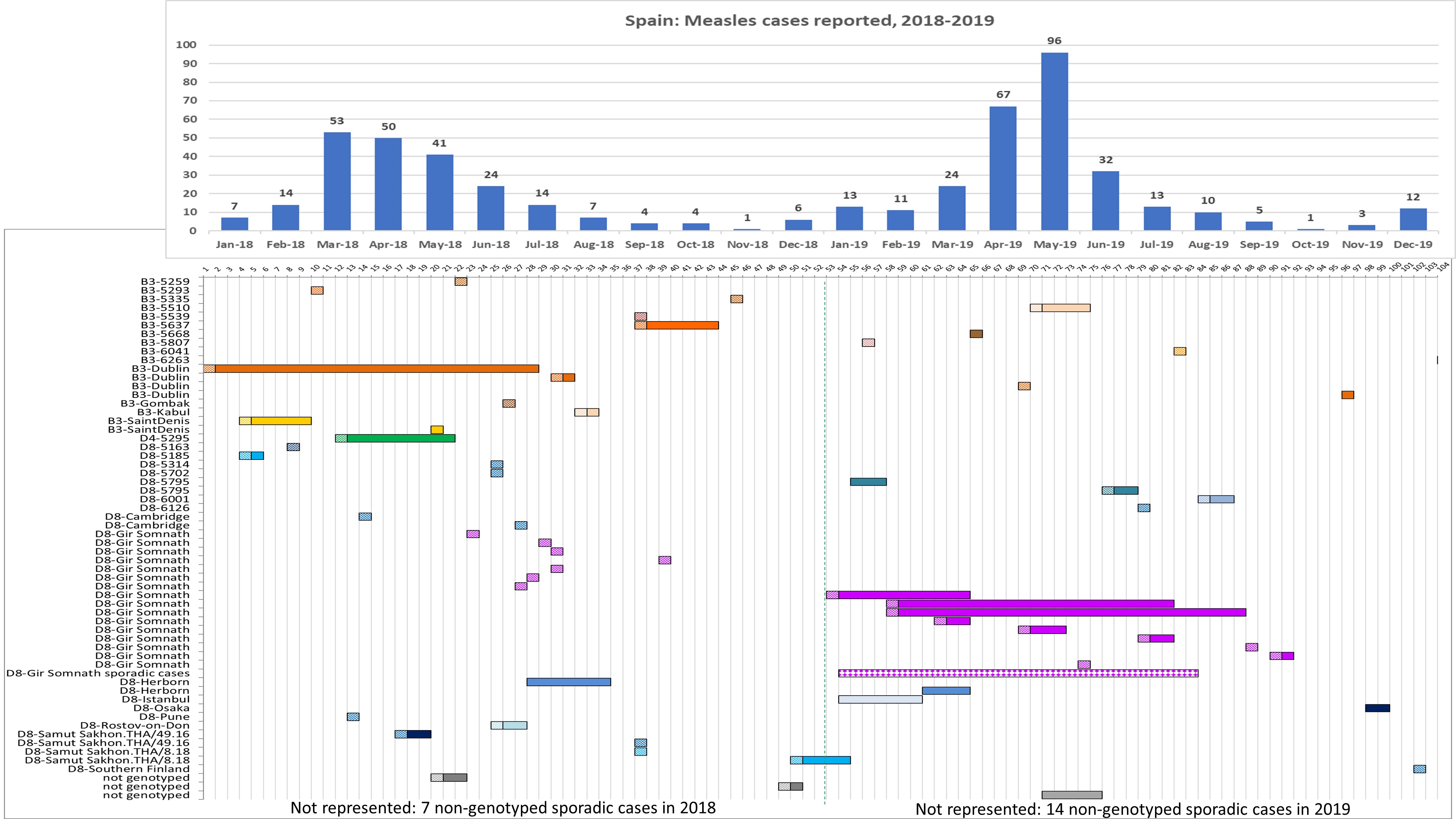

Supplement: Supplementary file 1 [file vaccines-12-00810-s001.zip › vaccines-3058263-supplementary.JPG]
